# Respiratory exacerbation in a young adult with cystic fibrosis and tricuspid atresia

**DOI:** 10.1002/rcr2.318

**Published:** 2018-04-01

**Authors:** Jamie Wood, Abbey Sawyer, Siobhain Mulrennan, Andrew Bullock

**Affiliations:** ^1^ Physiotherapy Department Sir Charles Gairdner Hospital Perth Australia; ^2^ Department of Respiratory Medicine Sir Charles Gairdner Hospital Perth Australia; ^3^ The Faculty of Health and Medical Sciences University of Western Australia Perth Australia; ^4^ Department of Cardiology ACHD Service, Sir Charles Gairdner Hospital Perth Australia

**Keywords:** Cystic fibrosis, exacerbation, Fontan, physiotherapy, tricuspid atresia

## Abstract

Tricuspid atresia (TAt) is a complex congenital heart defect (CHD) characterized by the absence of the tricuspid valve and right ventricular hypoplasia requiring surgery in childhood, the Fontan procedure. We present a case of a 21‐year‐old male with TAt and cystic fibrosis (CF), who underwent a Fontan procedure in childhood, presenting to an adult CF clinic with severe deterioration in his respiratory status and multi‐organ dysfunction associated with CF. This report describes problems associated with the management of a CF respiratory exacerbation and extrapulmonary manifestations of CF in the unique situation of a Fontan circulation, a circulation with absence of a subpulmonary ventricle and pulsatile pulmonary arterial blood flow where maintenance of systemic cardiac output is totally dependent on good respiratory function and low pulmonary artery pressures.

## Introduction

Tricuspid atresia (TAt) is a rare congenital heart defect (CHD), occurring in 0.5–1.2 per 10,000 live births 1. It is the third most common form of cyanotic CHD characterized by the absence of the tricuspid valve, absence of a direct connection between the right atrium and ventricle, right ventricular hypoplasia, atrial and ventricular septal defect (ASD, VSD), and severe pulmonary stenosis. In the absence of a subpulmonary ventricle, surgical correction to create a biventricular circulation is not possible. Surgical management of TAt involves creation of a unique circulation where the systemic venous (caval) return is redirected to the pulmonary arteries, the Fontan procedure. Maintenance of systemic cardiac output, exercise tolerance, and functional capacity is entirely “preload” dependent, that is, totally dependent on pulmonary blood flow. In the absence of a subpulmonary ventricle and pulsatile flow, pulmonary blood flow occurs by “passive” venous flow related to changes in intrathoracic pressure associated with breathing. Low pulmonary pressures and low transpulmonary gradient is essential to the function of the Fontan circulation 2. Cystic fibrosis (CF) is an autosomal recessive genetic condition associated with bronchiectasis, recurrent respiratory infection, deteriorating respiratory function and extrapulmonary manifestations 3. To date, there are no reports of a patient with TAt having undergone a Fontan procedure in the presence of CF.

We present a 21‐year‐old male with TAt and CF who, having undergone a Fontan procedure in childhood, presented to an adult CF clinic with severe respiratory and extrapulmonary dysfunction. This report describes the problems associated with management of CF in the unique situation of a patient with a Fontan circulation.

## Case Report

**Figure 1:**
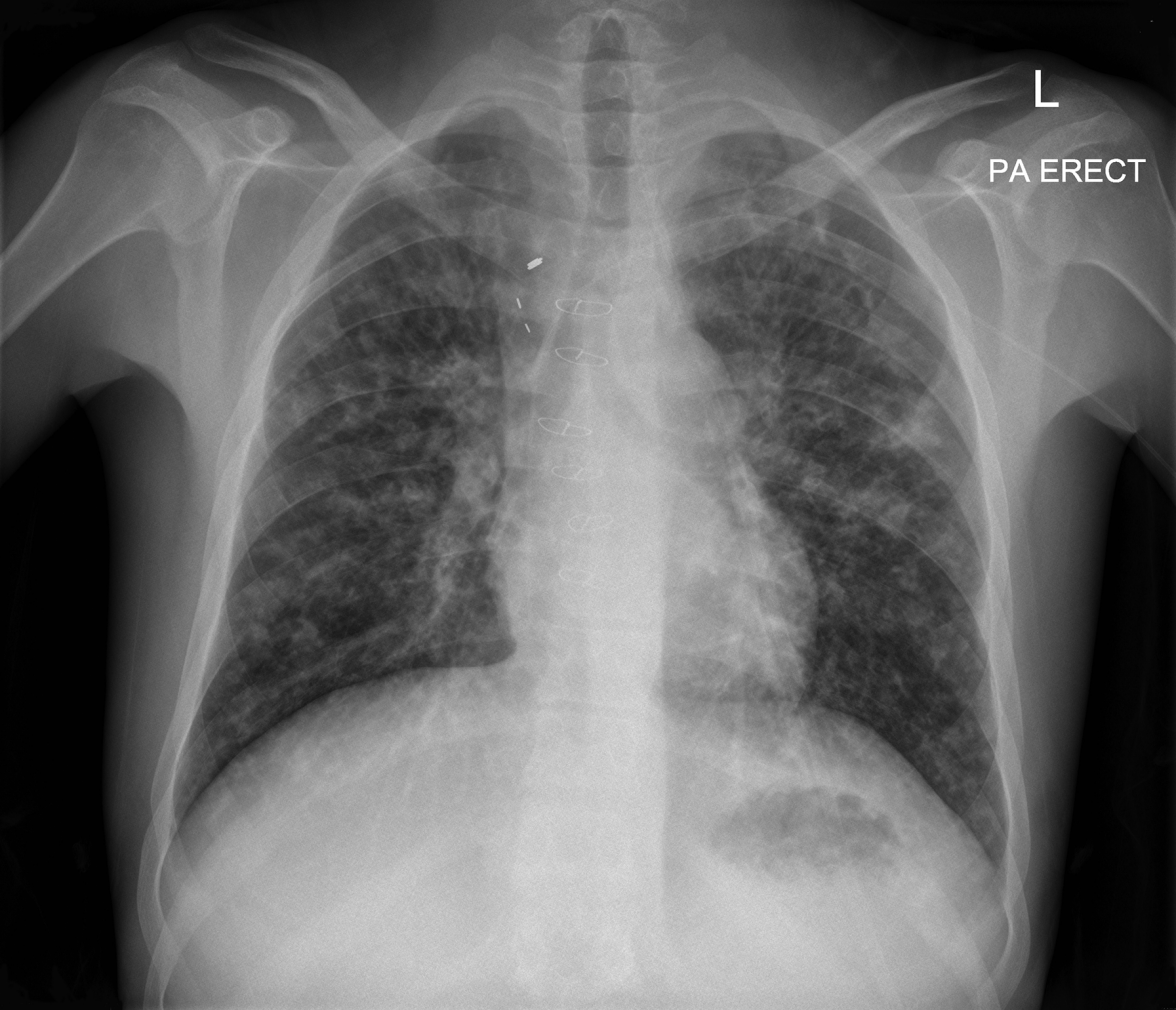
Chest radiography on admission demonstrating bilateral patchy consolidation on a background of bronchiectatic changes, with midline sternotomy wires and mediastinal clips from previous Fontan surgery.

A 21‐year‐old male with CF (homozygous for F508del mutation) was admitted to the Respiratory Medicine ward for 18 days with type 1 respiratory failure (arterial blood gas: pH 7.41, PaCO_2_ 43, PaO_2_ 54, HCO_3_ 26, BE 2) following a routine outpatient appointment at the CF centre. The patient had transitioned from the paediatric centre three years prior and had minimal contact with the adult CF or CHD service during this period. At presentation, his predicted forced expiratory volume in 1 s (ppFEV_1_) was 31% (previously 76%). Oxygen saturations (SpO_2_) were 81% on room air (RA) (previously 92%), temperature was 37.6°C, heart rate (HR) was 100 bpm, and blood pressure (BP) 130/80 mmHg. He had no dyspnoea at rest or with mild exertion. C‐reactive protein was 29 mg/L. Chest radiography (CXR) showed bilateral patchy consolidation (Fig. 1). Blood sugar levels on presentation ranged between 11 and 18 mmol/L but remained between 5 and 14 mmol/L after day 3 of admission.

Past medical history included complex CHD (functionally univentricular heart (FUVH): TAt, right ventricular hypoplasia, ASD, VSD, and severe pulmonary stenosis) for which he had undergone a two‐staged surgical and then a Fontan procedure (Total CavoPulmonary Connection with Extracardiac Conduit (ECC) aged 1, 2, and 8 years old, respectively). He had diabetes mellitus, liver cirrhosis, portal hypertension, oesophageal varices, splenomegaly, anaemia, osteopenia, vitamin deficiency, and substance abuse.

During the admission, he received intravenous antibiotics, and intensive medical and allied health input, including physiotherapy for airway clearance and exercise training. An Aerobika® (Trudell Medical International, London, Ontario, Canada) combined with saline or hypertonic saline was used for airway clearance, with low expiratory resistance used and HR and BP monitored carefully during initial sessions. Supplementary oxygen was provided to maintain SpO_2_ above 92%. After consultation with the adult congenital cardiologist, physiotherapists were advised to allow desaturation during exercise training and use patient‐reported dyspnoea when determining exercise intensity. The patient performed a 6‐min walk test (6MWT) on day 9 (8 L/min supplemental oxygen) and day 16 (on RA) (Table 1). Despite a clinically significant worsening in SpO_2_ during the second 6MWT, the 6MWT distance (6MWD) improved, and dyspnoea (Borg scale) remained unchanged. At discharge, the patient had a ppFEV_1_ of 42% and SpO_2_ of 84% (RA), which improved to a ppFEV_1_ of 51% and SpO_2_ was 88% 9 months post‐discharge.

**Table 1:** Six‐minute walk test results on days 9 and 16 of admission.

	Day 9	Day 16
Oxygen (L/min)	8	RA
Distance (m)	410	481
SpO_2_ pre (%)	95	84
SpO_2_ post (%)	90	78
Dyspnoea pre	0	0
Dyspnoea post	0.5	0.5
Peak HR (bpm)	130	133
Rest	Nil	Nil

SpO_2_, oxygen saturation; HR, heart rate; bpm, beats per minute. Dyspnoea measured using the Borg scale.

## Discussion

This case describes a very complex clinical presentation, involving a severe respiratory exacerbation and extrapulmonary manifestations of CF in the rare situation of a patient with a Fontan circulation. Patients with a Fontan circulation generally maintain resting SpO_2_ lower than the healthy population (e.g. 88–92%), with further desaturation permitted on exertion 4. The more marked systemic desaturation noted in this case was of some concern initially, albeit subjectively well tolerated, and oxygen therapy was initiated. Upon consultation with the congenital cardiologist, it became evident that desaturation was likely related to exacerbation in respiratory disease (as evidenced by the severely reduced ppFEV_1_) and intrapulmonary shunting and that oxygen therapy was unlikely to be of significant benefit. This contrasts with the usual recommendations for management of exacerbation of respiratory disease in CF 5. With improvement in respiratory status (ppFEV_1_) and exercise tolerance (6MWD), the patients’ SpO_2_ (at rest and with exercise) improved and eventually returned to the previous level. Physiotherapists’ were careful not to use high positive expiratory pressures during airway clearance as this may have reduced the forward flow of the Fontan circulation.

There are multiple other clinical features and potential complications associated with a Fontan circulation: arrhythmias, systemic thromboembolism, liver, renal and gastrointestinal abnormalities, polycythaemia, iron deficiency, anticoagulation and bleeding, and protein‐losing enteropathy (pleural and small bowel lymphangiectasia, hypoalbuminaemia, pleural effusions, ascites, plastic bronchitis) 1. In this young man, there are complex physiological and disease processes that impact the understanding and management of both CF and cardiac conditions. Given this patient’s complex medical history, early referral to the transplantation unit was initiated, such that in the event of significant deterioration in respiratory, cardiac, or functional status, the complex interplay of severe CF and a Fontan circulation might be better understood prior to consideration of combined heart, lung, and possibly liver transplantation.

This case report further highlights the increasing survival of individuals with CF, as with CHD, into adulthood and emphasizes the importance of effective transition from paediatric to highly specialized adult CF and CHD services if these improved outcomes are to be maintained into adulthood.

### Disclosure Statement

Appropriate written informed consent was obtained for publication of this case report and accompanying images.
